# (*E*)-*N*′-(3-Fluoro­benzyl­idene)-2-hydroxy­benzohydrazide

**DOI:** 10.1107/S1600536809006941

**Published:** 2009-03-06

**Authors:** Hua-Jie Xu, Liang-Quan Sheng, Zhao-Di Liu, Si-Chang Shao

**Affiliations:** a Department of Chemistry, Fuyang Normal College, Fuyang Anhui 236041, People’s Republic of China

## Abstract

The title compound, C_14_H_11_FN_2_O_2_, adopts an *E* or *trans* configuration with respect to the C=N bond. An intra­molecular N—H⋯O hydrogen bond contributes to the relatively planarity of the mol­ecular conformation; the two benzene rings are inclined to one another by 12.5 (2)°. In the crystal structure, inter­molecular O—H⋯O hydrogen bonds link the mol­ecules into chains running parallel to the *c* axis.

## Related literature

For the potential pharmacological and anti­tumor properties of hydrazones and Schiff bases, see: Karthikeyan *et al.* (2006 ▶); Khattab (2005 ▶); Kucukguzel *et al.* (2006 ▶); Okabe *et al.* (1993 ▶).
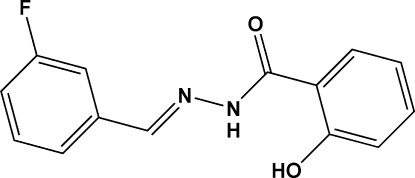


## Experimental

### 

#### Crystal data

C_14_H_11_FN_2_O_2_
*M* *_r_* = 258.25
Monoclinic, 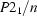

*a* = 4.8751 (15) Å
*b* = 22.188 (7) Å
*c* = 11.323 (3) Å
β = 96.717 (5)°
*V* = 1216.4 (6) Å^3^
*Z* = 4
Mo *K*α radiation
μ = 0.11 mm^−1^
*T* = 297 K
0.20 × 0.10 × 0.10 mm

#### Data collection

Bruker SMART CCD area-detector diffractometer
Absorption correction: multi-scan (*SADABS*; Sheldrick, 1996 ▶) *T*_min_ = 0.979, *T*_max_ = 0.989
8534 measured reflections
2152 independent reflections
1205 reflections with *I* > 2σ(*I*)
*R*_int_ = 0.081

#### Refinement

*R*[*F* ^2^ > 2σ(*F* ^2^)] = 0.088
*wR*(*F* ^2^) = 0.240
*S* = 1.05
2152 reflections
170 parameters
H-atom parameters constrained
Δρ_max_ = 0.20 e Å^−3^
Δρ_min_ = −0.30 e Å^−3^

### 

Data collection: *SMART* (Siemens, 1996 ▶); cell refinement: *SAINT* (Siemens, 1996 ▶); data reduction: *SAINT*; program(s) used to solve structure: *SHELXS97* (Sheldrick, 2008 ▶); program(s) used to refine structure: *SHELXL97* (Sheldrick, 2008 ▶); molecular graphics: *SHELXTL* (Sheldrick, 2008 ▶); software used to prepare material for publication: *SHELXTL*.

## Supplementary Material

Crystal structure: contains datablocks global, I. DOI: 10.1107/S1600536809006941/su2098sup1.cif

Structure factors: contains datablocks I. DOI: 10.1107/S1600536809006941/su2098Isup2.hkl

Additional supplementary materials:  crystallographic information; 3D view; checkCIF report

## Figures and Tables

**Table 1:** Hydrogen-bond geometry (Å, °)

*D*—H⋯*A*	*D*—H	H⋯*A*	*D*⋯*A*	*D*—H⋯*A*
N1—H1*N*⋯O1	0.86	1.91	2.612 (5)	138
O1—H1*O*⋯O2^i^	0.82	1.86	2.657 (5)	166

Symmetry code: (i) 
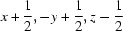
.

**Fig. 1.**
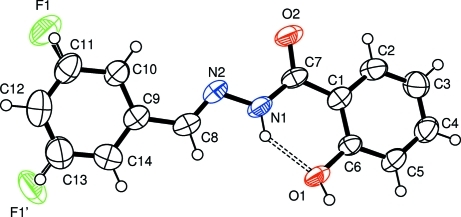
The molecular structure of the title compound, showing 30% probability displacement ellipsoids. H-atoms have been omitted for clarity.

**Fig. 2.**
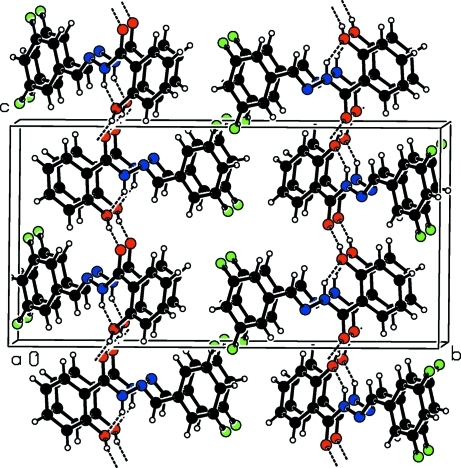
Crystal packing viewed along the A axis. The intra- and intermolecular hydrogen bonds are shown as dashed lines (details are given in Table 1).

## supplementary crystallographic information

### Comment

Hydrazones and Schiff bases have attracted much attention for their excellent biological properties, especially for their potential pharmacological and antitumor properties (Kucukguzel *et al.*, 2006; Khattab, 2005; Karthikeyan *et al.*, 2006; Okabe *et al.*, 1993). As part of an ongoing study of such compounds, we report here on the crystal structure of the title compound.

The molecular structure of the title compound is shown in Fig. 1. It can be seen to display a *trans* configuration about the C=N bond. There is an intramolecular N-H···O hydrogen bond, involving the NH H-atom and the O-atom of the hydroxyl substituent (Table 1), and the dihedral angle between the two benzene rings is 12.5 (2)°.

In the crystal molecules are linked by intermolecular O—H···O hydrongen bonds to form chains running parallel to the c axis (Fig. 2 and Table 1).

### Experimental

Equimolar amounts of 2-Hydroxybenzohydrazide and 3-fluorobenzohydrazide were reacted in ethanol (10 ml) for 1 h. After allowing the resulting solution to stand in air for 10 days yellow block-shaped crystals were formed, on slow evaporation of the solvent.

### Refinement

Phenyl ring C9-C14 was treated as a regular hexagon, and refined as a rigid body. The F-atom was found to be disordered over two positions, F1/F1', and given occupancies of 0.5/0.5. The H-atoms at C11 and C13 were also given occupancies of 0.5/0.5. All the H-atoms were placed in calculated positions  and treated as riding: O-H = 0.82 Å, N-H = 0.86 Å, C-H = 0.93 Å, with *U*_iso_(H) = 1.5*U*_eq_(parent O and N-atom, and = 1.2*U*_eq_(parent C-atom).

### Crystal data

**Table:**

C_14_H_11_FN_2_O_2_	*F*(000) = 536
*M**_r_* = 258.25	*D*_x_ = 1.410 Mg m^−^^3^
Monoclinic, *P*2_1_/*n*	Mo *K*α radiation, λ = 0.71073 Å
Hall symbol: -P 2yn	Cell parameters from 589 reflections
*a* = 4.8751 (15) Å	θ = 2.3–15°
*b* = 22.188 (7) Å	µ = 0.11 mm^−^^1^
*c* = 11.323 (3) Å	*T* = 297 K
β = 96.717 (5)°	Block, yellow
*V* = 1216.4 (6) Å^3^	0.20 × 0.10 × 0.10 mm
*Z* = 4	

### Data collection

**Table:**

Bruker SMART CCD area-detector diffractometer	2152 independent reflections
Radiation source: fine-focus sealed tube	1205 reflections with *I* > 2σ(*I*)
graphite	*R*_int_ = 0.081
φ and ω scans	θ_max_ = 25.1°, θ_min_ = 1.8°
Absorption correction: multi-scan (*SADABS*; Sheldrick, 1996)	*h* = −5→5
*T*_min_ = 0.979, *T*_max_ = 0.989	*k* = −26→26
8534 measured reflections	*l* = −13→13

### Refinement

**Table:**

Refinement on *F*^2^	Primary atom site location: structure-invariant direct methods
Least-squares matrix: full	Secondary atom site location: difference Fourier map
*R*[*F*^2^ > 2σ(*F*^2^)] = 0.088	Hydrogen site location: inferred from neighbouring sites
*wR*(*F*^2^) = 0.240	H-atom parameters constrained
*S* = 1.05	*w* = 1/[σ^2^(*F*_o_^2^) + (0.1113*P*)^2^ + 0.2945*P*] where *P* = (*F*_o_^2^ + 2*F*_c_^2^)/3
2152 reflections	(Δ/σ)_max_ = 0.002
170 parameters	Δρ_max_ = 0.20 e Å^−^^3^
0 restraints	Δρ_min_ = −0.30 e Å^−^^3^

### Special details

**Table:**

Geometry. Bond distances, angles etc. have been calculated using the rounded fractional coordinates. All su's are estimated from the variances of the (full) variance-covariance matrix. The cell esds are taken into account in the estimation of distances, angles and torsion angles
Refinement. Refinement of F^2^ against ALL reflections. The weighted R-factor wR and goodness of fit S are based on F^2^, conventional R-factors R are based on F, with F set to zero for negative F^2^. The threshold expression of F^2^ > 2sigma(F^2^) is used only for calculating R-factors(gt) etc. and is not relevant to the choice of reflections for refinement. R-factors based on F^2^ are statistically about twice as large as those based on F, and R- factors based on ALL data will be even larger.

### Fractional atomic coordinates and isotropic or equivalent isotropic displacement parameters (Å^2^)

**Table:**

	*x*	*y*	*z*	*U*_iso_*/*U*_eq_	Occ. (<1)
F1	−0.2554 (15)	0.0383 (3)	0.4844 (5)	0.103 (3)	0.500
F1'	−0.3673 (19)	0.0122 (3)	0.0916 (6)	0.136 (4)	0.500
O1	0.8661 (7)	0.26546 (15)	0.1078 (3)	0.0638 (11)	
O2	0.7116 (7)	0.27530 (14)	0.4613 (2)	0.0643 (11)	
N1	0.5969 (8)	0.23163 (16)	0.2828 (3)	0.0518 (12)	
N2	0.4125 (7)	0.19142 (17)	0.3234 (3)	0.0490 (12)	
C1	0.9330 (8)	0.31131 (19)	0.3001 (4)	0.0443 (12)	
C2	1.0667 (10)	0.3559 (2)	0.3713 (4)	0.0563 (16)	
C3	1.2548 (11)	0.3939 (2)	0.3302 (4)	0.0658 (19)	
C4	1.3165 (10)	0.3890 (2)	0.2158 (4)	0.0601 (17)	
C5	1.1869 (9)	0.34596 (19)	0.1418 (4)	0.0527 (17)	
C6	0.9959 (8)	0.30710 (19)	0.1824 (3)	0.0433 (14)	
C7	0.7399 (9)	0.27175 (19)	0.3545 (4)	0.0465 (17)	
C8	0.2866 (9)	0.1604 (2)	0.2415 (4)	0.0524 (17)	
C9	0.0844 (6)	0.11357 (12)	0.2623 (3)	0.0497 (17)	
C10	0.0213 (6)	0.09879 (13)	0.3754 (2)	0.0520 (16)	
C11	−0.1728 (7)	0.05426 (14)	0.3894 (3)	0.067 (2)	
C12	−0.3037 (6)	0.02449 (13)	0.2905 (4)	0.0750 (19)	
C13	−0.2406 (7)	0.03926 (15)	0.1774 (3)	0.083 (2)	
C14	−0.0466 (7)	0.08380 (16)	0.1634 (2)	0.073 (2)	
H1N	0.62100	0.23090	0.20870	0.0620*	
H1O	0.96210	0.25770	0.05500	0.0950*	
H2	1.02690	0.36010	0.44930	0.0680*	
H3	1.34130	0.42320	0.38030	0.0790*	
H4	1.44560	0.41470	0.18810	0.0720*	
H5	1.22770	0.34290	0.06380	0.0630*	
H8	0.32300	0.16740	0.16390	0.0630*	
H10	0.10890	0.11870	0.44160	0.0620*	
H11	−0.21500	0.04440	0.46510	0.0800*	0.500
H12	−0.43350	−0.00530	0.29990	0.0900*	
H13	−0.32820	0.01930	0.11120	0.0990*	0.500
H14	−0.00440	0.09370	0.08770	0.0870*	

### Atomic displacement parameters (Å^2^)

**Table:**

	*U*^11^	*U*^22^	*U*^33^	*U*^12^	*U*^13^	*U*^23^
F1	0.145 (6)	0.094 (5)	0.080 (4)	−0.033 (5)	0.054 (4)	0.006 (4)
F1'	0.184 (8)	0.138 (6)	0.075 (4)	−0.103 (6)	−0.026 (5)	−0.006 (4)
O1	0.076 (2)	0.075 (2)	0.0473 (19)	−0.0178 (19)	0.0363 (16)	−0.0120 (17)
O2	0.081 (2)	0.075 (2)	0.0416 (18)	−0.0068 (18)	0.0274 (17)	−0.0018 (15)
N1	0.063 (2)	0.056 (2)	0.041 (2)	−0.005 (2)	0.0255 (19)	0.0029 (18)
N2	0.051 (2)	0.053 (2)	0.047 (2)	0.003 (2)	0.0222 (18)	0.0074 (19)
C1	0.045 (2)	0.042 (2)	0.048 (2)	0.011 (2)	0.014 (2)	0.006 (2)
C2	0.074 (3)	0.053 (3)	0.044 (2)	0.001 (3)	0.016 (2)	−0.002 (2)
C3	0.080 (4)	0.053 (3)	0.064 (3)	−0.013 (3)	0.007 (3)	0.000 (3)
C4	0.061 (3)	0.062 (3)	0.059 (3)	−0.008 (3)	0.014 (2)	0.011 (3)
C5	0.061 (3)	0.050 (3)	0.051 (3)	−0.002 (2)	0.023 (2)	0.007 (2)
C6	0.047 (3)	0.045 (2)	0.040 (2)	0.004 (2)	0.014 (2)	−0.004 (2)
C7	0.054 (3)	0.048 (3)	0.041 (3)	0.013 (2)	0.021 (2)	0.001 (2)
C8	0.058 (3)	0.059 (3)	0.042 (3)	0.009 (3)	0.014 (2)	0.009 (2)
C9	0.052 (3)	0.047 (3)	0.052 (3)	0.009 (2)	0.014 (2)	0.005 (2)
C10	0.061 (3)	0.041 (2)	0.057 (3)	0.002 (2)	0.019 (2)	0.000 (2)
C11	0.076 (4)	0.049 (3)	0.080 (4)	0.001 (3)	0.031 (3)	0.006 (3)
C12	0.060 (3)	0.055 (3)	0.111 (4)	−0.004 (3)	0.014 (3)	0.008 (3)
C13	0.085 (4)	0.077 (4)	0.085 (4)	−0.017 (3)	0.004 (3)	0.001 (3)
C14	0.078 (4)	0.084 (4)	0.055 (3)	−0.012 (3)	0.003 (3)	−0.005 (3)

### Geometric parameters (Å, °)

**Table:**

F1'—C13	1.243 (8)	C9—C10	1.3908
F1—C11	1.243 (7)	C9—C14	1.3896
O1—C6	1.357 (5)	C10—C11	1.3899
O2—C7	1.236 (5)	C11—C12	1.3894
O1—H1O	0.8200	C12—C13	1.3908
N1—N2	1.383 (5)	C13—C14	1.3897
N1—C7	1.344 (6)	C2—H2	0.9300
N2—C8	1.256 (6)	C3—H3	0.9300
N1—H1N	0.8600	C4—H4	0.9300
C1—C7	1.474 (6)	C5—H5	0.9300
C1—C2	1.390 (6)	C8—H8	0.9300
C1—C6	1.405 (6)	C10—H10	0.9300
C2—C3	1.367 (7)	C11—H11	0.9300
C3—C4	1.368 (6)	C12—H12	0.9300
C4—C5	1.375 (6)	C13—H13	0.9300
C5—C6	1.386 (6)	C14—H14	0.9300
C8—C9	1.470 (5)		
			
C6—O1—H1O	109.00	C11—C12—C13	120.00
N2—N1—C7	122.6 (3)	C12—C13—C14	119.96
N1—N2—C8	112.9 (3)	F1'—C13—C12	117.6 (5)
C7—N1—H1N	119.00	F1'—C13—C14	122.5 (4)
N2—N1—H1N	119.00	C9—C14—C13	120.06
C6—C1—C7	125.3 (4)	C1—C2—H2	119.00
C2—C1—C7	117.4 (4)	C3—C2—H2	119.00
C2—C1—C6	117.3 (4)	C2—C3—H3	120.00
C1—C2—C3	122.0 (4)	C4—C3—H3	120.00
C2—C3—C4	120.3 (4)	C3—C4—H4	120.00
C3—C4—C5	119.7 (4)	C5—C4—H4	120.00
C4—C5—C6	120.6 (4)	C4—C5—H5	120.00
C1—C6—C5	120.2 (4)	C6—C5—H5	120.00
O1—C6—C1	119.9 (4)	N2—C8—H8	118.00
O1—C6—C5	120.0 (3)	C9—C8—H8	118.00
O2—C7—N1	121.6 (4)	C9—C10—H10	120.00
O2—C7—C1	121.4 (4)	C11—C10—H10	120.00
N1—C7—C1	117.0 (4)	C10—C11—H11	120.00
N2—C8—C9	123.2 (4)	C12—C11—H11	120.00
C10—C9—C14	119.98	C11—C12—H12	120.00
C8—C9—C14	117.4 (3)	C13—C12—H12	120.00
C8—C9—C10	122.6 (3)	C12—C13—H13	120.00
C9—C10—C11	119.97	C14—C13—H13	120.00
F1—C11—C10	126.5 (4)	C9—C14—H14	120.00
C10—C11—C12	120.04	C13—C14—H14	120.00
F1—C11—C12	113.4 (4)		
			
C7—N1—N2—C8	175.7 (4)	C4—C5—C6—O1	178.9 (4)
N2—N1—C7—O2	−0.8 (7)	C4—C5—C6—C1	−0.1 (6)
N2—N1—C7—C1	179.0 (4)	N2—C8—C9—C10	−1.7 (6)
N1—N2—C8—C9	178.6 (4)	N2—C8—C9—C14	178.1 (4)
C2—C1—C6—C5	0.8 (6)	C8—C9—C10—C11	179.7 (3)
C7—C1—C6—O1	2.7 (6)	C14—C9—C10—C11	−0.02
C7—C1—C6—C5	−178.3 (4)	C8—C9—C14—C13	−179.7 (3)
C2—C1—C7—O2	−4.8 (6)	C10—C9—C14—C13	−0.03
C2—C1—C7—N1	175.4 (4)	C9—C10—C11—F1	−177.2 (5)
C6—C1—C7—O2	174.3 (4)	C9—C10—C11—C12	0.03
C6—C1—C7—N1	−5.5 (6)	F1—C11—C12—C13	177.5 (4)
C2—C1—C6—O1	−178.2 (4)	C10—C11—C12—C13	−0.02
C6—C1—C2—C3	−0.8 (7)	C11—C12—C13—F1'	−178.6 (5)
C7—C1—C2—C3	178.3 (4)	C11—C12—C13—C14	−0.02
C1—C2—C3—C4	0.2 (7)	F1'—C13—C14—C9	178.5 (5)
C2—C3—C4—C5	0.5 (7)	C12—C13—C14—C9	0.03
C3—C4—C5—C6	−0.6 (7)		

### Hydrogen-bond geometry (Å, °)

**Table:**

*D*—H···*A*	*D*—H	H···*A*	*D*···*A*	*D*—H···*A*
N1—H1N···O1	0.86	1.91	2.612 (5)	138
O1—H1O···O2^i^	0.82	1.86	2.657 (5)	166

Symmetry codes: (i) *x*+1/2, −*y*+1/2, *z*−1/2.
